# Construction of a novel exosomes-related gene signature in hepatocellular carcinoma

**DOI:** 10.3389/fcell.2022.997734

**Published:** 2022-08-29

**Authors:** Qiqi Tao, Kai Zhu, Yating Zhan, Rongrong Zhang, Zhichao Lang, Zhengping Yu, Meng Wang

**Affiliations:** Department of Hepatobiliary Surgery, The First Affiliated Hospital of Wenzhou Medical University, Wenzhou, China

**Keywords:** hepatocellular carcinoma, exosome, prognostic signature, immune infiltration, nomogram

## Abstract

**Background:** Exosomes are extracellular vesicles between 40 and 150 nm in diameter and are cargoes for a wide range of small biological molecules. Recent studies have reported that lncRNAs, miRNAs, circRNAs in serum exosomes may serve as biomarkers to predict hepatocellular carcinoma (HCC) prognosis. However, the prognostic values of exosomes-related mRNAs in HCC are still unclear.

**Methods:** Data of HCC patients were downloaded from The Cancer Genome Atlas (TCGA) database. The serum exosome sequencing data of HCC patients and healthy individuals were obtained from the exobase database. Univariate cox regression analysis was used to identify prognostic exosomes-related genes. LASSO and multivariate cox regression analyses were applied to construct prognostic signature.

**Results:** 22 exosomes-related mRNAs differentially expressed between HCC tissues and normal tissues were identified. Then, 8 prognostic exosomes-related mRNAs were screened. Subsequently, G6PD and ADAMTS5, selected by LASSO and multivariate cox regression analyses, were used to construct a prognostic signature. The patients with high-risk scores had a poor prognosis in TCGA cohort as well as ICGC cohort. Notably, this prognostic signature was also validated in a local cohort collected from the First Affiliated Hospital of Wenzhou Medical University. Receiver Operating Characteristic (ROC) analyses indicated that the signature had a good performance in all the cohorts. The gene set enrichment analysis revealed that this signature was associated with cell cycle and metabolism pathways. Immune infiltration analysis indicated that the patients with high-risk scores had a higher M0 macrophages infiltration. The univariate and multivariate cox regression analyses identified that the risk score is an independent risk factor for HCC. In addition, a nomogram containing age, gender, stage and risk score was constructed to precisely predict HCC prognosis.

**Conclusion:** In conclusion, we develop a novel exosomes-related gene signature that helps to predict HCC prognosis.

## Introduction

Hepatocellular carcinoma (HCC), the leading cause of cancer-related deaths worldwide, is one of the most common solid malignancies (Villanueva 2019). Despite advances in prevention, screening and new diagnostic and treatment techniques, treatment of HCC has encountered bottlenecks. The 5-year survival rate for patients with HCC remains below 20%, indicating that liver cancer remains a highly fatal disease (Jemal et al., 2017). The search for new prognostic markers for HCC is therefore of great importance.

Exosomes are extracellular vesicles between 40 and 150 nm in diameter (Kowal et al., 2014). Exosomes carry a wide range of cellular molecules including proteins, DNA, lipids, mRNA, miRNA, lncRNA, etc., and almost all cell types could secrete exosomes (Théry et al., 2002; Kahlert and Kalluri 2013; Raposo and Stoorvogel 2013). In recent years, increasing evidence has shown that exosomes are important carriers of specific signals in physiological scenarios (Becker et al., 2016). Exosomes have been demonstrated to promote HCC progression via multiple signaling pathways (Wang et al., 2018; Li et al., 2019; Zhang et al., 2020). Exosomes harbor small molecules that mediate immune regulation in the HCC microenvironment to shape the tumor microenvironment in which HCC develops (Wu et al., 2019). Several studies have shown that exosomal lncRNAs, miRNAs, circRNAs in serum exosomes have potential as biological markers of HCC prognosis (Xu et al., 2018; Wang et al., 2019; Yu et al., 2019). However, the role of exosomes-related mRNAs in HCC prognosis is largely unexplained. In this study, an exosomes-related gene signature was constructed for the prognosis of HCC patients, which provides a better understanding of the prognosis prediction.

## Methods and Materials

### Data sources

Serum exosome sequencing data, including lncRNA, mRNA, and circRNA sequencing data, were downloaded from the exobase database (http://www.exorbase.org/) for HCC patients (*n* = 112) and healthy individuals (*n* = 118). Transcriptome sequencing data and clinical follow-up data of HCC were downloaded from The Cancer Genome Atlas Program (TCGA) (*n* = 371) and International Cancer Genome Consortium (ICGC) (*n* = 231) databases. In addition, we collected 100 surgically resected tissues from patients with HCC admitted to the First Hospital of Wenzhou Medical University and performed transcriptome sequencing as the local cohort. This study involving human participants were reviewed and approved by the Human Research Ethics Committee of the First Affiliated Hospital of Wenzhou Medical University. And all participants signed a written informed consent form. Specific clinical parameters for the TCGA cohort, ICGC cohort and local cohort were shown in Table 1.

**TABLE 1:** Specific clinical parameters for the TCGA, cohort, ICGC, cohort and local cohort.

Clinical parameters	Variable	TCGA cohort	ICGC cohort	Local cohort
age	<=65	230 (61.99%)	89 (38.53%)	32 (32.00%)
	>65	141 (38.01%)	142 (61.47%)	68 (68.00%)
gender	FEMALE	120 (32.35%)	61 (26.41%)	30 (30.00%)
	MALE	251 (67.65%)	170 (73.59%)	70 (70.00%)
grade	G1	55 (14.82%)	0 (0.00%)	16 (16.00%)
	G2	178 (47.98%)	0 (0.00%)	43 (43.00%)
	G3	120 (32.35%)	0 (0.00%)	33 (33.00%)
	G4	13 (3.5%)	0 (0.00%)	5 (5.00%)
	unknown	5 (1.35%)	231 (100.00%)	3 (3.00%)
stage	Stage I	174 (46.9%)	36 (15.58%)	51 (51.00%)
	Stage II	85 (22.91%)	105 (45.45%)	26 (26.00%)
	Stage III	84 (22.64%)	71 (30.74%)	21 (21.00%)
	Stage IV	4 (1.08%)	19 (8.23%)	1 (1.00%)
	unknown	24 (6.47%)	0 (0.00%)	1 (1.00%)

### Competing endogenous RNA network construction and differentially expressed genes screening

Serum exosome sequencing data were downloaded from the exobase database for HCC and healthy individuals. The R package “limma” was used to identified different expressions of mRNA, lncRNA, and circRNA between HCC patients and healthy individuals. Then the miRNAs which may interact with these differentially expressed RNAs were predicted by TargetScan, miRanda, miRcode, starBase databases. According to the “ceRNA hypothesis”, a lncRNA-miRNA-mRNA-circRNA network was constructed (Salmena et al., 2011). Cytoscape software (v3.8.2) was used to visualize the ceRNA network. The mRNAs from the ceRNA network were included in the subsequent analysis. Then differential analysis between HCC tissues and normal tissues was performed to identify differentially expressed exosomes-related genes (DEEGs). Gene Ontology (GO) enrichment analysis was performed on DEEGs.

### Exosomes-related genes prognostic signature construction

Differentially expressed mRNA expression data from the TCGA database were combined with survival data, and then univariate cox regression analysis was performed to screen prognosis-related DEEGs. The Least absolute shrinkage and selection operator (LASSO) algorithm was applied to prognosis-related DEEGs to remove overfitting model genes. Finally, multivariate cox regression analysis was performed to derive biased regression coefficients for each gene in the signature. The risk score was calculated as Risk score = $e^{\overset{N}{\underset{i=1}{\sum }}\left(\text{Exp}\left(\text{i}\right)\cdot \text{coe}\left(\text{i}\right)\right)}$ , where Exp(i) is the expression of the genes (G6PD and ADAMTS5) in the signature and coe(i) is the bias regression coefficient of the genes derived from the multivariate cox regression analysis. All HCC patients were separated into low- and high-risk group based on the median value of risk score.

### Validation of prognostic exosomes-related genes signature

Survival curves on low- and high-risk group were plotted by Kaplan-Meier methods for the TCGA cohort, ICGC cohort, and local cohort using the “survival” and “survminer” packages. Principal component analyses (PCA) were performed to test the effectiveness of dichotomous classification of low- and high-risk subgroup patients using the R package “ggplot2”. Receiver Operating Characteristic (ROC) analyses were performed to verify the prognostic accuracy of the signature for patients with HCC in the first, second and third years, and the area under the ROC (AUC) was calculated using the R package “timeROC".

### Gene set enrichment analysis, tumor mutation burden, immune cell infiltration analysis

Firstly, we screened the TCGA cohort for differentially expressed genes between high- and low-risk subgroups, and then the packages “limma”, “org.Hs.eg.db”, “DOSE”, “clusterProfiler”, and “enrichplot” were used to perform the GSEA on these differentially expressed genes. The tumor mutation data for the high- and low-risk patients of the TCGA cohort were compiled using Perl software, and the results were visualized using the R package “maftools”. For immune cell infiltration analysis, we used the CIBERSORT algorithm to calculate the 22 type immune cell scores for each HCC patient in the TCGA cohort, and the R package “pheatmap” was used to plot the heat map of 22 immune cell relative infiltration content. The R packages “reshape2”, “ggpubr”, and “limma” were used to draw box-line plots of 22 type immune cell relative infiltration.

### Independent prognostic analysis, nomogram construction and calibration plots

To screen out prognosis-related clinical indicators, univariate and multivariate cox analysis was performed among the clinical characteristics including age, gender, grade, stage, T-stage and risk score in the TCGA cohort. In addition, we analyzed the correlation relationship between risk score and clinical characteristics using “limma” and “ggpubr” R packages. The R package “rms” was used to construct nomogram based on age, gender, stage, and risk score. Calibration curves were plotted to verify the accuracy of the nomogram in predicting prognosis.

### Statistical analysis

Statistical analyses in this study were performed by R software (V4.10). Student’s t test and oneway ANOVA were used to separately perform the group comparisons of two subgroups and more than two subgroups. The Kaplan-Meier method was used for survival analyses in this study. All statistical analyses were considered statistically significant only if the *p* < 0.05.

## Results

### Identification of differential exosomes-related genes

**FIGURE 1:**
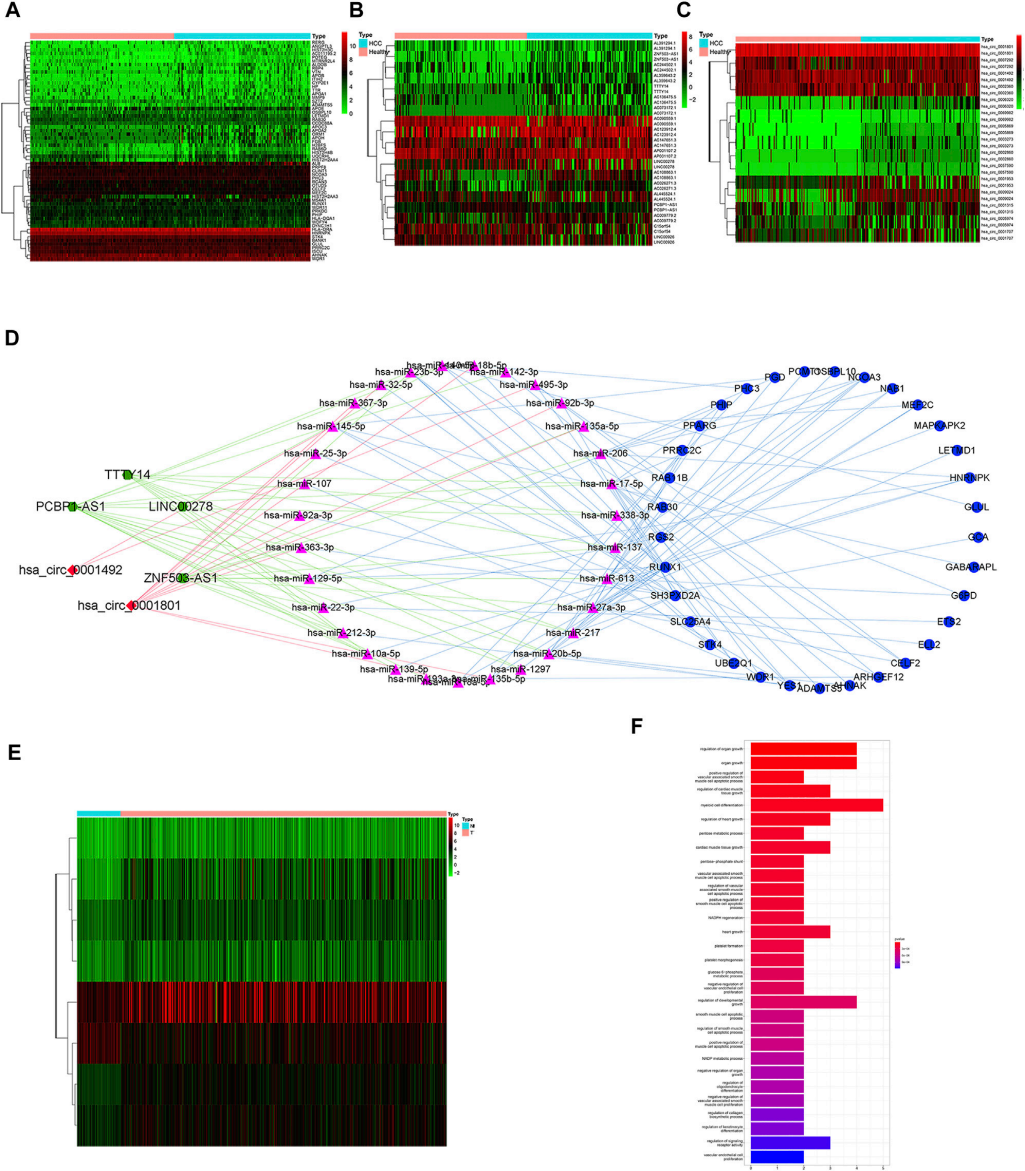
Identification of differential exosomes-related genes **(A)** heatmap of differentially expressed mRNAs between HCC patients and healthy individuals. **(B)** heatmap of differentially expressed lncRNAs between HCC patients and healthy individuals. **(C)** heatmap of differentially expressed circRNAs between HCC patients and healthy individuals. **(D)** ceRNA network; Green hexagon nodes represent lncRNAs, red diamond nodes represent circRNAs, red triangle nodes represent miRNAs, and blue round nodes represent mRNAs. **(E)** heatmap of differentially expressed exosomes-related mRNAs from ceRNA network between HCC tissues and adjacent normal tissues. **(F)** barplot of GO enrichment analysis for DEEGs.

Differential analysis of mRNAs, lncRNAs and circRNAs in serum exosomes was performed between 112 HCC patients and 118 healthy individuals (Figures 1A–C). We screened 134 mRNAs, 19 lncRNAs and 15 circRNAs. Then we predicted miRNAs that may bind to them and constructed a ceRNA network (Figure 1D). The ceRNA network included 33 mRNAs, 4 lncRNAs, 31 miRNAs and 2 circRNAs. Exosomes-related mRNAs from ceRNA network were performed differential analysis in the TCGA cohort and 22 DEEGs were identified (Figure 1E). The results of GO enrichment analysis showed that the DEEGs were mainly enriched in myeloid cell differentiation, regulation of organ growth, organic growth, DNA-binding transcription factor binding and cadherin binding pathways (Figure 1F).

### Construction of a 2-DEEG prognostic signature

**FIGURE 2:**
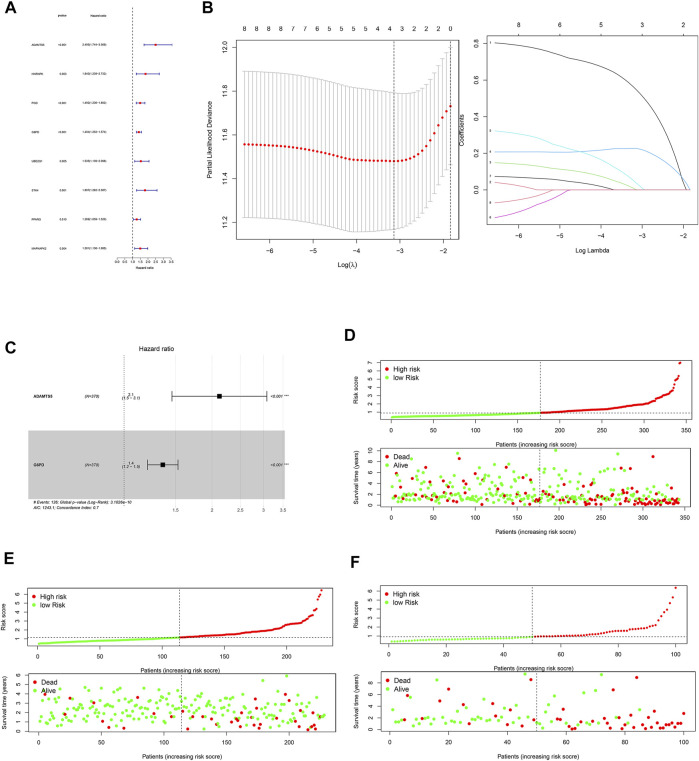
Construction of a 2-DEEG prognostic signature. **(A)** forest plot of univariate regression analysis. **(B)** lasso regression analysis of prognosis-related DEEGs. **(C)** forest plot of multivariate regression analysis. **(D)** survival scatter plot and risk score curve of TCGA cohort. **(E)** survival scatter plot and risk score curve of ICGC cohort. **(F)** survival scatter plot and risk score curve of local cohort.

8 prognostically exosomes-related genes were screened by univariate cox regression analysis (Figure 2A). All the 8 prognostically exosomes-related genes were considered as high-risk genes with hazard ratios >1, meaning the high expression of these genes related with the poor prognosis of HCC patients. ADAMTS5 and G6PD were identified to construct the prognostic signature by Lasso and multivariate cox regression analyses (Figures 2B,C). The risk score was calculated as: Risk score= e^ ((ADAMTS5 exp * 0.75) + (G6PD exp * 0.30)). The risk score was obtained for each patient according to the risk score calculation formula. Patients from TCGA cohort, ICGC cohort, and local cohort were classified into high- and low-risk subgroups based on the median risk score of each cohort, respectively. Survival status scatter plots showed that patients in high-risk subgroup had a shorter survival in comparison with low-risk group (Figures 2D–F).

### Validation of the prognostic 2-DEEG signature

**FIGURE 3:**
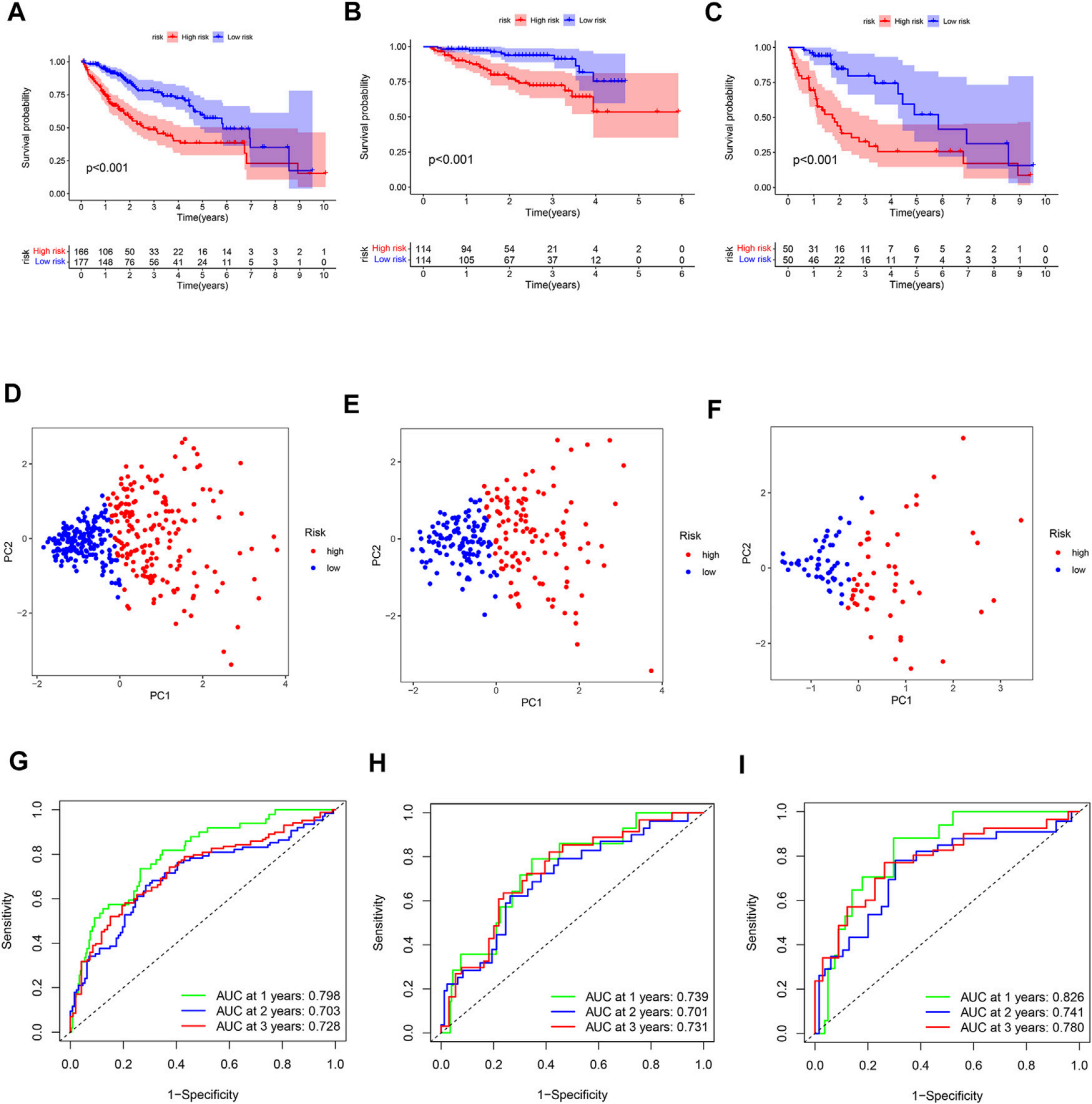
Validation of the prognostic 2-DEEG signature. **(A)** survival curve of TCGA cohort between high- and low-risk subgroups. **(B)** survival curve of ICGC cohort between high- and low-risk subgroups. **(C)** survival curve of local cohort between high- and low-risk subgroups. **(D)** PCA plot of TCGA cohort based on risk score. **(E)** PCA plot of ICGC cohort based on risk score. **(F)** PCA plot of local cohort based on risk score. **(G)** ROC curve of TCGA cohort. **(H)** ROC curve of ICGC cohort. **(I)** ROC curve of local cohort.

Survival analyses showed that patients in the high-risk subgroup had a poor prognosis (Figures 3A–C). The PCA plot indicated that the patients with different risk were distributed in two directions (Figures 3D–F). The AUC value of the ROC reached 0.798 at 1-year, 0.703 at 2-year, and 0.728 at 3-year in the TCGA cohort (Figure 3G). Moreover, the AUC values of ICGC cohort were 0.739, 0.701, and 0.731 for 1-year, 2-year, and 3-year, respectively (Figure 3H). The 1-year, 2-year, and 3-year AUC values of the local cohort were 0.826, 0.741, and 0.780, respectively (Figure 3I). The above results indicate that the 2-DEEG prognostic signature has a good performance in the prediction of HCC prognosis.

### GSEA enrichment, tumor mutation burden and immune cell infiltration analyses

**FIGURE 4:**
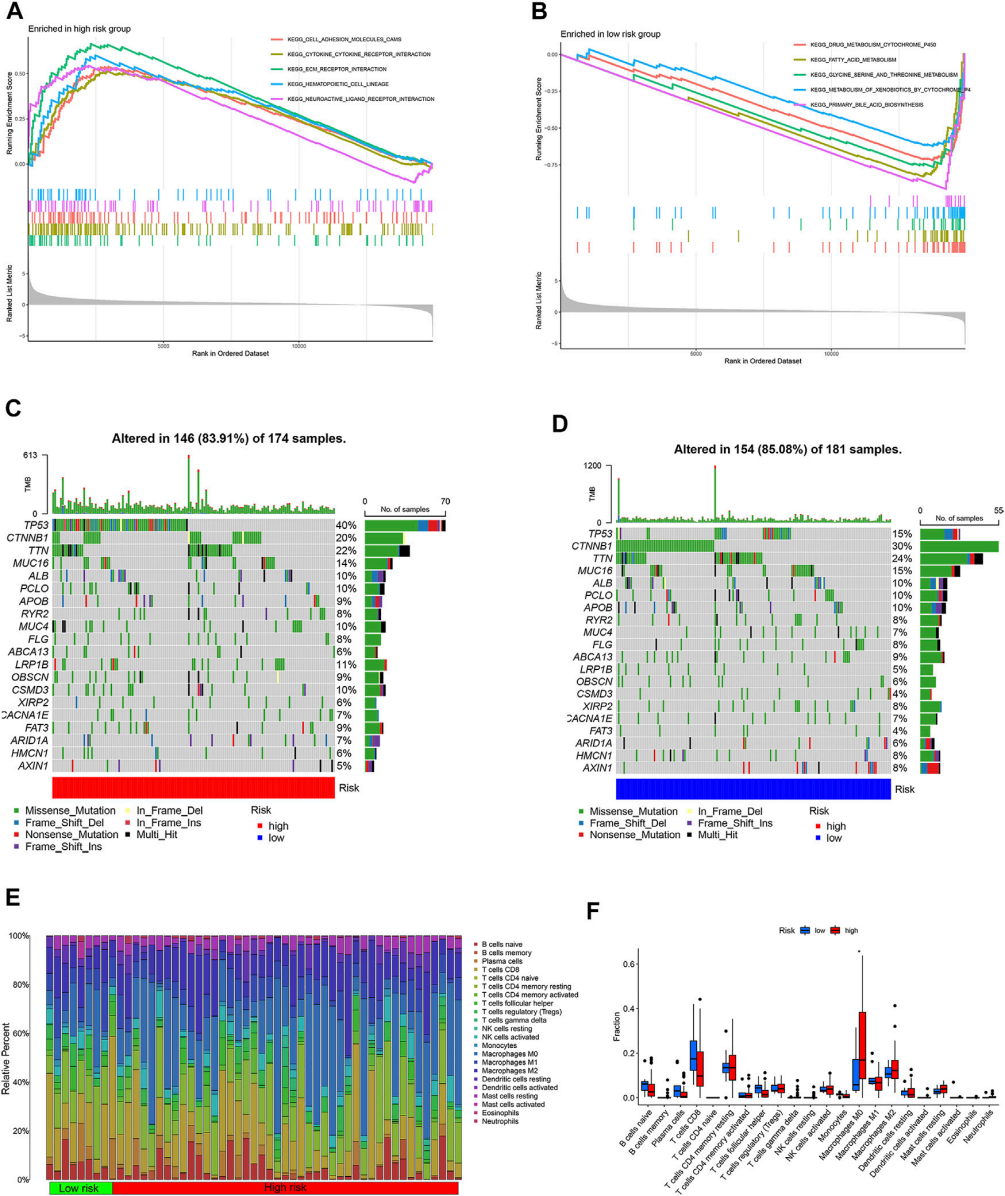
GSEA enrichment, tumor mutation burden and immune cell infiltration analyses. **(A)** GSEA enrichment analysis of high-risk subgroup. **(B)** GSEA enrichment analysis of low-risk subgroup. **(C)** tumor mutation burden waterfall plot of high-risk subgroup. **(D)** tumor mutation burden waterfall plot of low-risk subgroup. **(E)** heatmap of 22 type immune cells relative infiltration content. **(F)** difference analysis of 22 type immune cells relative infiltration content between high- and low-risk subgroup.

The differentially expressed mRNAs were identified between high- and low-risk subgroups in the TCGA cohort. GSEA enrichment analysis showed that upregulated mRNAs in the high-risk subgroup were mainly enriched in cell cycle and the interaction of cells (Figure 4A). Metabolism pathway such as fatty acid metabolism, drug metabolism was considered to enrich in low-risk subgroup (Figure 4B).

The tumor mutation burden waterfall plot showed the mutation rate of TP53 in the high-risk subgroup was higher than low-risk subgroup patients. Conversely, the CTNNB1 mutation rate was lower in the high-risk subgroup patients (Figures 4C,D). CIBERSORT algorithm was used to calculate immune cell scores for each HCC patient in the TCGA cohort. The immune cell relative content of each HCC patients was shown in Figure 4E. Interestingly, high risk score was associated with M0 macrophages (Figure 4F).

### Clinical stratified survival analysis

**FIGURE 5:**
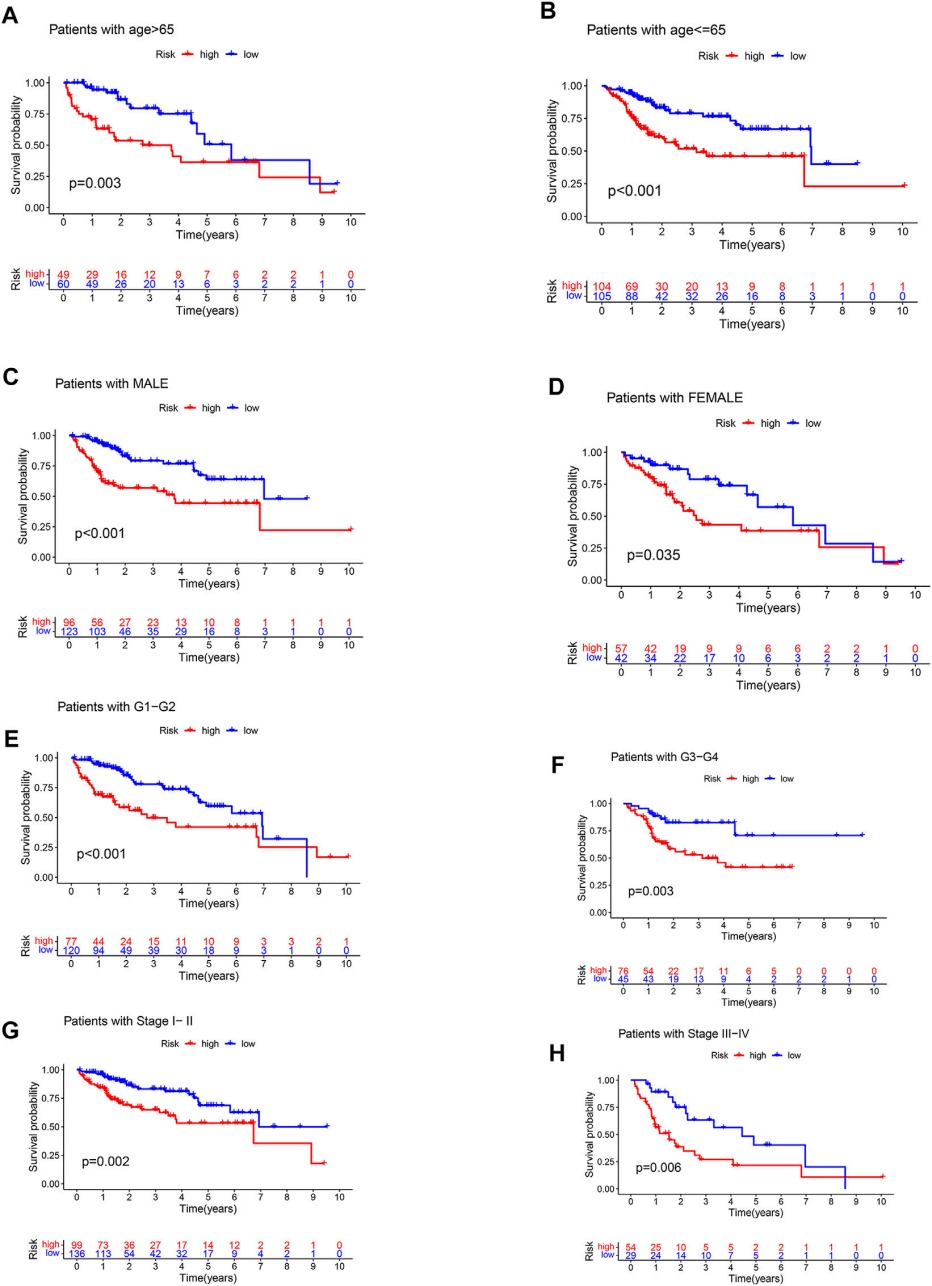
Clinical stratified survival analysis. **(A)** survival analysis of patients with age >65. **(B)** survival analysis of patients with age <=65. **(C)** survival analysis of male patients. **(D)** survival analysis of female patients. **(E)** survival analysis of patients with grade 1–2. **(F)** survival analysis of patients with grade 3–4. **(G)** survival analysis of patients with stage I-II. **(H)** survival analysis of patients with stage III-IV.

The TCGA cohort of HCC patients was divided into different clinical subgroups according to clinical characteristics: age (age>65 or <=65), gender (male or female), grade (grade 1-2, or grade 3-4), stage (stage I-II or stage III-IV), and T stage (T1-2 or T3-4). Survival analysis showed that OS was lower in patients with high-risk in the all clinical subgroups (Figure 5).

### Risk score is an independent prognostic factor for hepatocellular carcinoma

**FIGURE 6:**
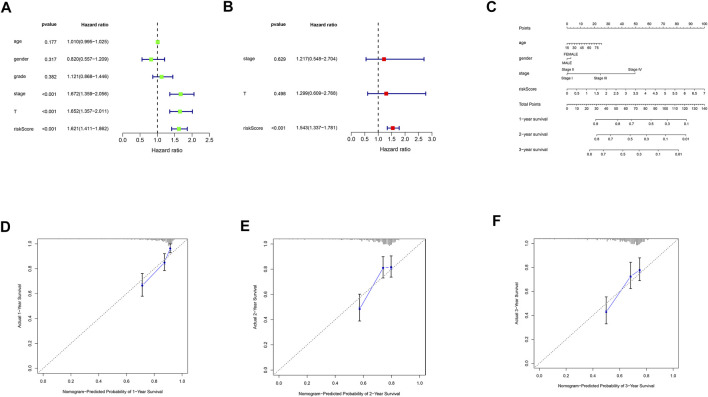
Nomogram construction and calibration plots. **(A)** univariate cox regression analysis of clinical characteristics and risk score. **(B)** multivariate cox regression analysis of prognosis-related clinical characteristics and risk score. **(C)** nomogram based on age, gender, stage and risk score. **(D)** calibration plot of the nomogram for the first year. **(E)** calibration plot of the nomogram for the second year. **(F)** calibration plot of the nomogram for the third year.

Univariate and multivariate cox analyses demonstrated that risk score was an independent prognostic factor for HCC (Figures 6A,B). Correlation analyses revealed that risk score was associated with grade and stage in HCC patients (Supplementary Figure S1). We then constructed a nomogram based on age, gender, stage, and risk score for precisely predicting HCC prognosis (Figure 6C). The calibration curves were used to verify the accuracy of the nomogram (Figures 6D–F).

## Discussion

Biological molecules in exosomes have been reported to promote proliferation, migration, and invasion of HCC. For instance, Fang et al. elucidated that tumor derived exosomal miR-1247-3p fosters lung metastasis of liver cancer through inducing cancer-associated fibroblast activation (Fang et al., 2018). CircCMTM3 derived from HCC cells exosomes promotes angiogenesis and tumorigenesis of HCC through miR-3619-5p/SOX9 axis (Hu et al., 2021). Furthermore, some exosome-derived biological molecules have been proved to be potential prognostic biomarkers for HCC. Yokota et al. reported that expression of serum exosomal miR-638 has the potential to serve as a significant and independent prognostic marker for HCC (Yokota et al., 2021). Noncoding RNAs deriving from circulating exosomes may be prognostic biomarkers in HCC (Lee et al., 2019). Herein, we explored the prognostic values of exosomes-related genes in HCC.

In this study, we constructed an HCC prognostic signature consisting of exosomes-related genes (G6PD and ADAMTS5). Upregulated G6PD promotes HCC progression through several pathways. Zhang et al. found that TSP50-induced cell proliferation and tumor formation were mediated by G6PD K171 acetylation (Zhang et al., 2021). In addition, miR-122 and miR-1 have been reported to inhibit HCC progression by mediating the inhibition of G6PD expression to suppress the pentose phosphate pathway (Barajas et al., 2018). Currently, the role of ADAMTS5 in HCC progression has not been elucidated. A recent study has shown that ADAMTS5 rs2380585 genotype is associated with the risk of HCC (Li et al., 2015). Unfortunately, there are few studies on the role of exosome-derived G6PD and ADAMTS5 in the progression of HCC.

In addition to the signature, a novel ceRNA network was revealed in this study. In our constructed ceRNA network, G6PD is a target gene of hsa-miR-206 and ADAMTS5 is a target gene of has-miR-212-3p. Hsa-miR-206 has been shown to inhibit HCC progression through multiple pathways (Chang et al., 2018; Hu et al., 2020; Lin et al., 2022). Wang et al. found that miR-206 could inhibit lipid accumulation and growth of hepatocellular carcinoma cells by targeting G6PD (Wang et al., 2021). Chen et al. found that miR-212-3p could inhibit HCC proliferation and invasion by suppressing CTGF expression (Chen et al., 2019). Exosome-derived G6PD and ADAMTS5 may regulate HCC progression via hsa-miR-206 and hsa-miR-212-3p, respectively. More experiments are needed to perform to validate the relevant molecular mechanisms of G6PD and ADAMTS5 in HCC in the future.

Mutations in CTNNB1, which encodes β-Catenin, result in the stabilization, nuclear translocation, and activation of the Wnt/β-Catenin cascade. Importantly, gain of function mutations of CTNNB1 gene could be found in ∼15–30% of HCC (Perugorria et al., 2019). In this study, the low-risk subgroup had a high CTNNB1 mutation rate and a better prognosis. Liang et al. reported that CTNNB1 mutant induces TBX3 suppressing HCC growth by inactivating PDL-1 (Liang et al., 2021). Our results were consistent with the previous findings. Senni et al. found that β-catenin participates in fatty acid oxidation metabolic reprogramming in HCC (Senni et al., 2019). The GSEA enrichment analysis showed the upregulated mRNAs in the low-risk subgroup enriched in the fatty acid metabolism, suggesting a key role of fatty acid metabolism in the low-risk subgroup. Taken together, inhibition of fatty acid oxidation may be a suitable therapeutic approach for CTNNB1-mutated HCC.

Our study has many advantages. Firstly, a 2-DEEG prognostic signature was constructed, which contributes to a better prediction of HCC prognosis. The prognostic values of this signature were validated in ICGC cohort and local cohort. Subsequently, a new nomogram including age, gender, stage, and risk score was developed for precisely predicting HCC prognosis. However, this study also has some limitations. The accuracy of the signature was needed for further validation in HCC. Moreover, *in vitro* and *in vivo* experiments were demanded to validate our results.

## Conclusion

In conclusion, we develop a novel exosomes-related gene signature that helps to predict the prognosis.

## Data Availability

The original contributions presented in the study are included in the article/Supplementary Material; further inquiries can be directed to the corresponding authors.

## Supplementary material

Supplementary Figure S1 Correlation analyses between risk score and different clinical subgroups **(A)** correlation analyses between risk score and different age subgroups. **(B)** correlation analyses between risk score and different gender subgroups. **(C)** correlation analyses between risk score and different grade subgroups. **(D)** correlation analyses between risk score and different stage subgroups.
