# Supplementary figures and images for: Construction of a novel exosomes-related gene signature in hepatocellular carcinoma
Source: Front Cell Dev Biol. 2022 Aug 29;10:997734. doi: 10.3389/fcell.2022.997734 (PMC9465081; doi:10.3389/fcell.2022.997734)

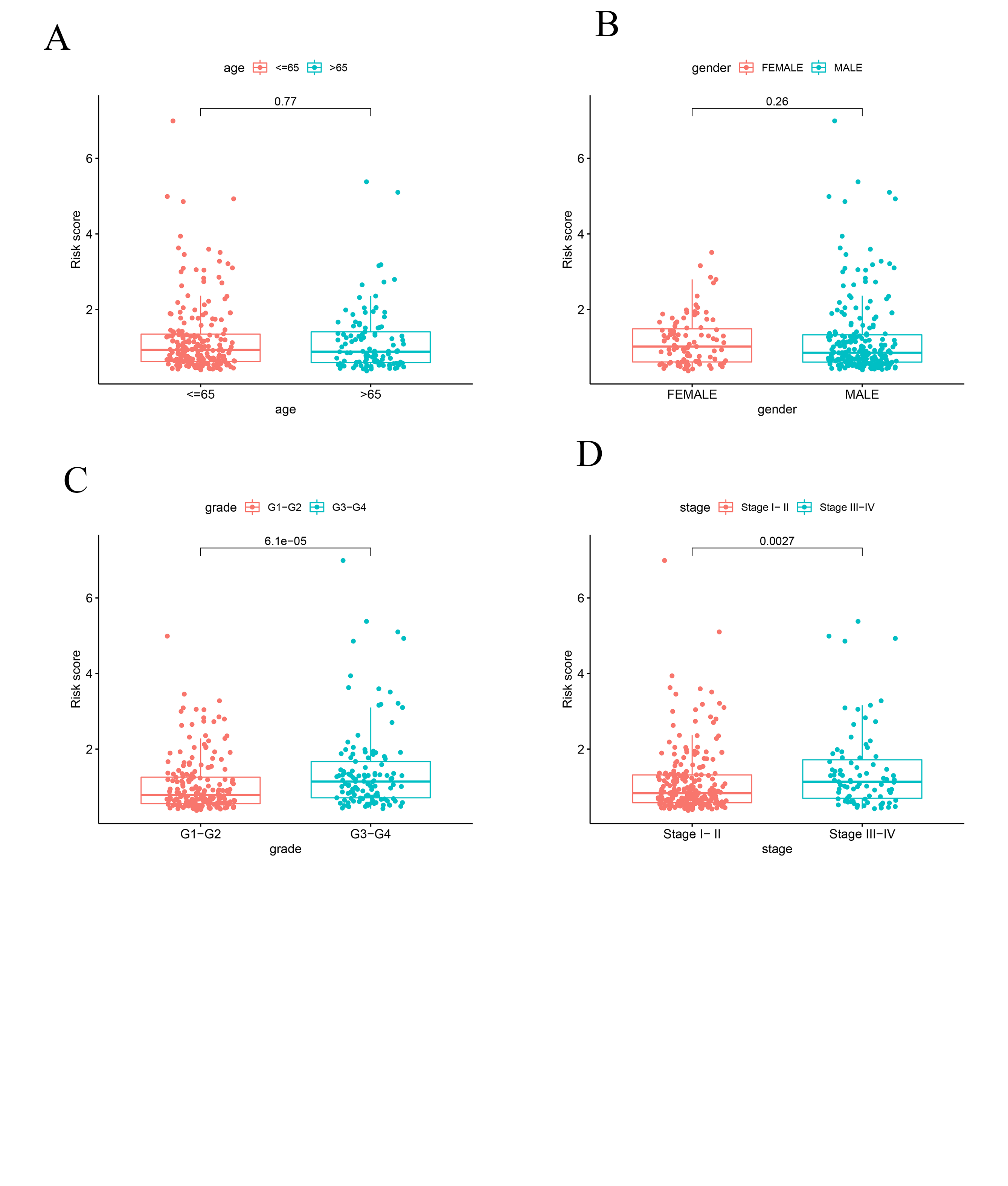

Supplement: Supplementary file 2 [file Image1.TIF]
